# Automatic speech analysis combined with machine learning reliably predicts the motor state in people with Parkinson’s disease

**DOI:** 10.1038/s41531-025-00959-4

**Published:** 2025-05-02

**Authors:** Tabea Thies, Elisa Mallick, Johannes Tröger, Ebru Baykara, Doris Mücke, Michael T. Barbe

**Affiliations:** 1 University of Cologne, Faculty of Medicine and University Hospital Cologne, Department of Neurology, Cologne, Germany; 2 University of Cologne, Faculty of Arts and Humanities, IfL Phonetics, Cologne, Germany; 3 ki:elements GmbH, Saarbrücken, Germany

**Keywords:** Diagnostic markers, Parkinson's disease

## Abstract

It is still under debate whether levodopa treatment improves speech functions in Parkinson’s disease (PD). Therefore, speech functions of people with PD were compared in medication-OFF condition (withdrawal of PD medication for at least 12 h) and medication-ON condition (after receiving 200 mg of soluble levodopa). A total of 78 participants, including 51 males and 27 females, performed predefined standard speech tasks. Acoustic speech features were automatically extracted with the algorithm given by the Dysarthria Analyzer. Results suggest that acute levodopa intake improves phonatory-respiratory speech functions and speech planning abilities, while the articulatory system remains unaffected. Furthermore, the study provided preliminary evidence that speech function is able to predict the medication status in individuals with PD as the constructed speech-based biomarker score did not only correlate with established measures of (speech) motor impairment but could also differentiate between the medication OFF and ON status. A post-hoc machine learning model yielded similar results.

## Introduction

Levodopa is an effective treatment for improving motor performance in Parkinson’s disease (PD)^1^. However, the exact effect of levodopa on speech motor control is under debate. It is still under debate how the different subsystems of speech production are changed in neurodivergent speech production, and how to quantify the changes in the acoustic domain related to respiration, phonation, and articulation in a reliable way. The respiratory-phonatory subsystem involves characteristics related to the air flow that passes from the lungs to the vocal cords (respiration), causing them to vibrate and creating voiced sounds (phonation) and speech melody to convey linguistic meaning. The articulatory system involves the movements of the articulators related to syllable production. This includes tongue tip and tongue dorsum, upper and lower lip, velum and the jaw to distinguish between the different sound qualities of consonants and vowels in a language system. By extracting reliable acoustic features as measurable properties the speech performance can be described, assessed, and monitored. In this regard, acoustic features of speakers are related to perceived parameters of listeners. For example, intensity corresponds to how loud a produced sound is perceived by the listener, while fundamental frequency corresponds to the perception of voice pitch.

Previous studies that investigated levodopa effects on acoustic speech features report inconsistent findings. Whereas some studies found increased intensity^2^, better voice control^3^, faster articulation rates of syllable production^4^ (i.e. faster, louder and more sonorous speech), others found no change regarding pitch^4–10^, articulation/speech rate^5,6,10,11^, and intensity^4,10^. With regard to vowel space changes, smaller vowel spaces^12^ but also larger vowel spaces^13^ (only in a subgroup of people with PD^14^) were observed. Note that larger vowel spaces are related to a more distinct and clear articulation. Two recent studies propose that only respiratory-phonatory features of speech are responsive to levodopa, while articulatory features are not^15,16^. This might indicate that acoustic features related to breath and vocal cord control, such as intensity and voice-related characteristics (e.g., pitch modulation, voice quality), change under levodopa, while the movement control of oral articulatory patterns for consonant and vowel productions do not (e.g., vowel frequencies, vowel durations).

With respect to the automated screening of levodopa-related speech performance changes, predicting motor status (medication-ON versus medication-OFF) is a binary classification problem. However, considering previous work, automatic detection of motor status seems possible. One study determined that phonatory speech features improve after levodopa intake^17^. By combining acoustic voice features, such as jitter, shimmer, and harmonics-to-noise ratios that were automatically extracted from phoneme production (/a, o, m/), a support vector machine classifier was able to differentiate a bad motor status (medication-OFF) from a good motor status (medication-ON) with an AUC of 0.81. Changes in the sound production of /o/ and /m/ were reported to be most sensitive to quantify levodopa-related voice changes.

Another study also investigated speech features related to voice functions automatically extracted from sustained phonation of the vowel /e/^18^. In line with the first study, voice features changed under levodopa indicating that phonatory control improved while articulatory features in terms of vowel frequencies reflecting tongue body configurations seem to be less levodopa-responsive. The authors further highlight that classic machine learning models perform better in differentiating medication conditions compared to convolutional neural networks. However, both studies included non-speech tasks, i.e. sustained vowel phonation. Only one study automatically derived speech features of natural speech tasks, such as reading or storytelling, highlighting that classifier results are highly dependent on the speech task^19^. Natural speech tasks seem more sensitive in accurately defining medication condition and motor status respectively.

Acoustic (automatic) speech feature analyses can be used as a promising tool to analyze speech performance changes in PD. Assessing speech performance can have several advantages for clinical implications, such as monitoring overall motor status of the people with PD (PwPD), determining subtle changes in motor control that are also manifested in speech performance, or assessing the response to levodopa. Continuous speech analyses offer the opportunity to track fluctuations clinicians might be interested in, to gain insights into the effectiveness of levodopa on motor control over time to improve personalizing treatment plans. As speech is critical for communication and maintaining social interaction, integrating (automatic) speech analysis into clinical routine, healthcare providers can focus on quality-of-life improvements for PwPD by addressing not just gross motor symptoms, but also the often-overlooked impact of speech impairments on daily functioning and social engagement.

However, it remains a challenge to detect levodopa effects on speech as there are no established tools that can be used in clinical practice to determine clinically relevant speech changes in PD so far. To date, no study proved the applicability of a comprehensive automatic analysis for measuring short-term effects of levodopa treatment on acoustic speech features. Thus, in this paper, we investigate levodopa-related speech changes in a large and well-characterized cohort of PwPD. Speech changes will be characterized in terms of analytical and clinical validation (Table 1). For clinical validation, we report group comparisons based on fitted linear mixed models between single speech features and our established speech-based composite score and whether they change depending on the medication status (med-OFF vs med-ON). Further, associations between single speech features as well as the composite score with the total motor score assessed with the ‘Unified Parkinson’s Disease Rating Scale’ (UPDRS III) will be presented. For analytical validation, we report associations between single speech features and the composite score with the UPDRS III.

**Table 1:** Overview over analysis process

	Analytical validation	Clinical validation	
Single Speech Features	Correlation UPDRS III item 18	Correlation UPDRS III total score	Group Comparison (med-OFF vs med-ON)
Composite Score	Correlation UPDRS III item 18	Correlation UPDRS III total score	Group comparison (med-OFF vs med-ON)
Machine Learning			ML Classification

Validation will be performed at three levels of granularity: 1) single speech features, 2) a composite score built from multiple acoustic speech features using conventional statistics and 3) an algorithm to automatically differentiate between medication ON and OFF state using machine learning and single speech features. Thus, this study is a step to an automated speech analysis that contributes to a comprehensive screening of motor status in PD via automatic speech assessment to use speech as a sensitive indicator of motor function in the future.

## Results

### Motor assessment

Descriptive statistics of the motor assessment results are depicted in Table 2. The linear mixed models reveal a significant effect of levodopa on the total UPDRS III score [X^2^(1) = 162.63, *p* < 0.001] and the speech score [X^2^(1) = 32.472, *p* < 0.001]. The mean difference of the total UPDRS III score was 15.8 points and of the speech item 0.4 points respectively.

**Table 2:** Results motor assessment in medication OFF and medicationON condition

UPDRS III		med-OFF	med-ON
total score	Mean (sd)	37 (10)	21 (8)
	Range	16–60	7–42
speech item	Mean (sd)	0.85 (0.94)	0.42 (0.63)
	Range	0–3	0–3

### Levodopa effect on single speech features

An overview over single speech features that significantly differed between both treatment conditions is given in Table 3 (left column). The mean difference and the p-values given from the post-hoc analyses are reported. In the following, the statistical results will be outlined in more detail for significant comparisons only.

**Table 3:** Results of post-hoc group comparisons between single speech features that significantly differed between both medication conditions as well as correlation coefficients between UPDRS III scores and single speech features

	group comparison		correlation with UPDRS III total score		correlation with UPDRS III speech item	
	difference	*p* value	coefficient	*p* value	coefficient	*p* value
DDKI (DDK)	5.77	0.031	–	n.s.	–	n.s.
VOT (DDK)	0.85	0.032	0.23	0.043	–	n.s.
stdPWR (DDK)	0.30	0.010	0.30	0.007	–	n.s.
DPI (monologue)	19.7	0.016	0.29	0.01	0.33	0.003
jitter (phonation A)	0.079	0.022	0.26	0.021	0.27	0.018
shimmer (phonation A)	0.405	0.033	0.23	0.038	–	n.s.
RST (text)	10.6	0.018	0.24	0.031	–	n.s.
stdPWR (text)	−0.202	0.001	−0.36	0.002	−0.43	<0.001
stdF0 (text)	–	n.s.	−0.23	0.04	−0.39	<0.001

Non-significant results are indicated by n.s.

For the oral diadochokinesis task (DDK), statistical analyses revealed that VOT differed significantly between OFF and ON condition with smaller values in the ON condition, the mean difference was 0.951 [OFF: 28.9 ± 5.7, ON: 28.0 ± 4.67, *t*(79) = 2.188, *p* = 0.032]. Further, DDKI significantly diverged between both conditions [OFF: 62.6 ± 33, ON: 56.8 ± 26, t(79) = 2.196, *p* = 0.031]. In addition, stdPWR was higher in the OFF condition compared to the ON condition [OFF: 3.1 ± 1.3, ON: 2.8 ± 1.1, *t*(79) = 2.626, *p* = 0.010].

Statistical analyses of features extracted from the monologue revealed that DPI significantly differed between OFF and ON condition [OFF: 305 ± 121, ON: 285 ± 113, *t*(79) = 2.455, *p* = 0.016]. Values are smaller in the ON condition.

The phonation of the vowel /a/ significantly differed between OFF and ON condition, as the values of the jitter [OFF: 0.58 ± 0.36, ON: 0.50 ± 0.20, *t*(79) = 2.335, *p* = 0.022] and shimmer features [OFF: 3.46 ± 2.92, ON: 3.06 ± 2.30, *t*(79) = 2.168, *p* = 0.033] decreased.

Two features that were extracted from the reading tasks, significantly differed between OFF and ON condition, namely RST [OFF: 389 ± 63, ON: 378 ± 60, *t*(79) = 2.411, *p* = 0.018] and stdPWR [OFF: 3.34 ± 0.69, ON: 3.54 ± 0.73, *t*(79) = −3.339, *p* = 0.001]. While values of the RST decreased, values of the stdPWR increased in the ON condition.

### Composite score building: correlations of speech features and UPDRS scores

Each extracted speech feature was correlated with the UPDRS III total score. Correlation coefficients and *p*-values are reported in Table 3 (middle column). With the exception of the DDKI feature, every feature found in the group comparisons also correlates with the UPDRS III total score. In addition, the stdF0 feature extracted from the reading text correlates with the UPDRS III total score.

Each extracted speech feature was also correlated with the UPDRS speech score. Correlation coefficients and *p*-values are reported in Table 3 (right column). As can be seen, only four features correlate with the UPDRS III speech score. These are DPI, jitter as well as stdPWR and stdF0 extracted from the reading text.

### Composite score validation

**Fig. 1:**
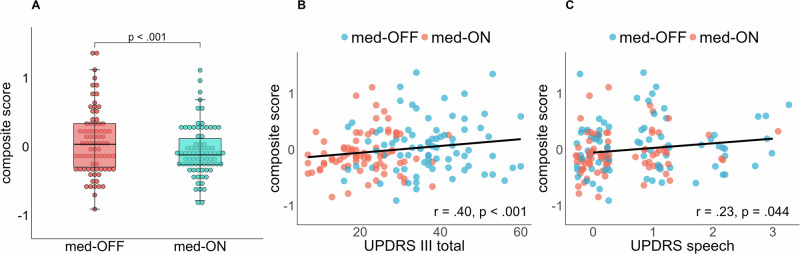
Composite score analysis across medication conditions and its correlation with UPDRS III measures. **A** Group comparison of composite score between both medication conditions. **B** Correlation between UPDRS III total score and created composite score. **C** Correlation between UPDRS III speech score and created composite score. Jitter was added along the x-axis to increase readability of the data.

The composite score was constructed from all features listed in Table 3 except for the DDKI feature and the stdF0 extracted from the text. As shown in Fig. 1A, the composite score could significantly differentiate between the medication conditions [X² = 12.909, *p* < 0.001].

The clinical validation of the constructed composite score showed a significant moderate correlation with the established UPDRS III total score (*r* = 0.40, *p* < 0.001, Fig. 1B). The result was proven by fitting a linear mixed effect model. The effect of the UPDRS III total score on the composite score was significant [β = 0.007, SE = 0.002, *t*(100.80) = 3.872, *p* < 0.001].

The analytical validation of the constructed composite score showed a significant low correlation with the speech item 18 of the UPDRS III (*r* = 0.23, *p* = 0.044, Fig. 1C). The result was also proven by the fitted linear mixed effect model, as the effect of the UPDRS III speech score on the composite score was significant [β = 0.09, SE = 0.04, *t*(151.77) = 2.424, *p* = 0.0165].

### Machine learning

**Fig. 2:**
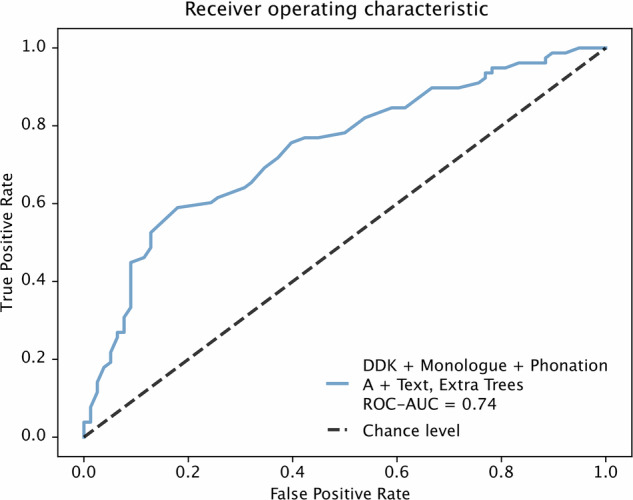
Results of ROC analysis of the linear model considering all recorded speech tasks. Age and sex were regressed out from the dataset using a linear mixed effect model beforehand.

The machine learning experiment showed the best classifier with a ROC-AUC of 0.74 using all tasks’ features and feature selection to classify between medication OFF and ON condition (Fig. 2).

## Discussion

This study captured acute levodopa effects on acoustic speech features in a large cohort of PwPD. Findings indicate that particularly phonatory and respiratory as well as speech timing patterns improve under levodopa.

Phonatory changes were observable in terms of voice quality (jitter, shimmer) revealing reduced hoarseness during sustained phonation of the vowel /a/. This indicates a change in voice quality due to improved vocal fold control as it has been shown before^3^.

As the loudness variation was reduced in the oral diadochokinesis (DDK) task, the production of fast syllable repetitions is stabilized under levodopa. For the reading task, an increase in loudness variation was found indicating more vivid speech production and mitigation of monoloudness under levodopa^10^. Thus, the control over the respiratory system improved under levodopa as it has been observed before^5,20^. In parallel, there is also an increase in fundamental frequency variation indicating a reduction of monopitch. Both phenomena can possibly be explained based on reduced rigidity and hypokinesia of the relevant musculature.

In addition, articulatory timing in the DDK task was improved as the variation decreased, further highlighting more stable DDK production. The reading text was produced with a slower articulation rate reducing acceleration of speech tempo and rushes of speech. In addition, fewer difficulties initiating speech and shorter pause durations during the picture description tasks indicate that dopamine is involved in speech motor preparation and execution. The production of precise temporal coordination patterns in the syllable-internal consonant-vowel production was also found to change under levodopa^20,21^.

No changes were detected in articulatory features of oral vocal tract movements, such as vowel duration and articulation rate. This fits the recent assumption that speech subsystems respond differently to levodopa^22^. Although the VOT feature of stop consonants in the fast syllable repetition task /pa-ta-ka/ has been found to differ between medication conditions. However, this variation in the timing of phonation relative to the release of a stop consonant in the oral vocal tract is not particularly meaningful and should be interpreted with caution.

To summarize, the articulatory system remains rather unaffected by levodopa, while the phonatory-respiratory system benefits from acute levodopa intake and seems to depend on dopaminergic brain circuits.

Interestingly, diadochokinetic irregularity (DDKI) was the only feature that significantly differed between medication conditions but that did not correlate with the measure of motor impairment severity (UPDRS III). DDKI refers to the standard deviation of the measured durations between consecutive voice onsets in a fast syllable repetition task of /pa-ta-ka/. The mean value in med-OFF status seems comparable to what was reported by Hlavnička for severely affected people with PD^23^. As the value decreases under levodopa, the pace of alternating motion rates improves. Inappropriate timing in fast syllable repetitions (as captured by DDKI) might not be directly linked to aspects of motor impairment assessed with the UPDRS III as the assessment primarily evaluates the amplitude and speed of the movement rather than stability of movement repetitions over a longer period of time.

Our findings underscore the utility of a composite score that distinguishes between medication conditions in PwPD. The significant differentiation between medication ON and OFF condition confirms the score’s sensitivity to changes in motor status, indicating its potential as a reliable biomarker for monitoring therapeutic effects^24,25^.

The composite score also displayed a moderate correlation with the established UPDRS III total score which further substantiates the composite score’s relevance in clinical settings. This correlation suggests that the composite score not only reflects levodopa-induced changes but also aligns well with a widely accepted clinical measure of motor function severity. Such alignment reinforces the composite score’s potential to complement existing clinical assessments, providing a more nuanced understanding of patient status.

The results from the machine learning experiment highlights feasibility of using speech features alongside an automatic decision support algorithm in classifying medication OFF and ON conditions. While recent advances in deep learning for speech analysis are promising, our feature engineering approach remains more appropriate given our relatively small sample size, which is insufficient for robust deep learning models. Additionally, composite scores offer greater interpretability, which is crucial in clinical settings where transparency is key for decision-making. From a regulatory standpoint, methods like ours may also be easier to validate and approve for clinical trials or medical devices. In the future, integrating more advanced techniques like deep learning could be valuable, but will require larger datasets and further validation.

Despite the promising results, these results have several limitations. Firstly, the motor status was evaluated only in a binary fashion (medication ON versus medication OFF) without considering the severity of motor impairment. This binary approach may overlook the nuances of motor function changes, limiting the ability to fully capture the spectrum of disease progression and treatment response. It would be interesting to investigate whether the composite score could also predict general motor function, but a larger dataset is needed for this. Nevertheless, it can be summarized that the OFF/ON analysis also has its relevance, as, for example, in the application of closed-loop therapy systems, it would be clinically very relevant to determine the patient’s current motor state through speech.

As data was collected by means of a levodopa challenge test, the order of test conditions was fixed and not randomized. However, this test is not only a standard assessment in clinical practice, also speech data has been collected during such assessments in many previous studies^26–28^. While a randomized sequence of medication conditions would be ideal, it is not logistically feasible in a clinical setting. The fixed order of medication administration may lead to practice or fatigue effects, which could contribute to the observed medication effects. If fatigue had occurred during the second assessment, it would have systematically reduced performance on the subsequent speech task, thereby enhancing rather than diminishing the observed levodopa effect. Furthermore, conducting tests on different days would necessitate a medication withdrawal period of at least 12 h in between, potentially introducing additional confounding effects as it is also essential to recognize that speech function can be influenced by various factors, including sleep quality, time of day, and the participant’s daily status or feelings—elements that are inherently challenging to control. To enhance the rigor of future studies and better account for confounding effects, the inclusion of a placebo and control group should be considered.

Thirdly, the study lacks validation through a second dataset, which would enhance the generalizability of the findings. Validation using an independent dataset is crucial to confirm the robustness and applicability of the composite score across different patient populations. As we only included participants that responded to levodopa therapy, future analyses need to explore if the results are reproducible if also non-responders were included.

Additionally, the study did not assess perceptual factors such as speech intelligibility^29–31^, which could provide a more comprehensive understanding of the impact of speech impairment on daily life. Intelligibility, as a measure of how well speech is understood by listeners, is directly relevant to patients’ communication abilities and overall quality of life. Including such measures in future research would offer a more holistic view of the functional consequences of motor impairments. However, as previous studies have shown, particularly measures of vocalic contrast and vowel space as well as consonant production correlate with intelligibility^32–34^, which were not assessed in this study, so that future investigation needs to test whether intelligibility could be predicted from our data. In addition, the relation between other acoustic features and intelligibility prediction are complex^35,36^, another study showed that feature sets of each of phonatory, articulatory, and prosodic subsystems could discriminate intelligibility at least in a binary fashion^37^.

Finally, although our study shares methodological similarities with the research conducted by Tykalová et al.^10^, our findings differ, and it is important to discuss potential reasons for these discrepancies. Both studies assessed the short-term effects of levodopa following comparable withdrawal periods and utilized identical speech tasks, analyzing the same acoustic features. The motor impairment levels in both cohorts were similar; however, our study included both early and mid-stage PwPD, resulting in slightly higher impairment levels. Since levodopa responsiveness decreases and motor complications increase with disease progression, this difference is unlikely to account for variations in study outcomes. One potential factor contributing to the differing results is the type of microphone used as Tykalová et al. employed an omnidirectional microphone, while we used a cardioid microphone. Omnidirectional microphones capture sound equally from all directions, whereas cardioid microphones are most sensitive to sounds from the front, with sensitivity varying based on placement. To control for placement effects, we maintained a consistent distance between the mouth and microphone across recording sessions by using a headset microphone. Cardioid microphones are designed to isolate the main sound source while minimizing background noise. Therefore, we do not anticipate that microphone choice significantly affected the extracted features. Importantly, our study exclusively included PwPD who exhibited a motor response to levodopa, applying a higher response threshold (30%) compared to Tykalová et al.’s (20%). Adhering to guideline-recommended thresholds might have led them to observe similar results. Language differences could also influence outcomes, as German and Czech belong to different branches of the Indo-European language family (Germanic vs. West Slavic). While most extracted features are not strongly affected by language, differences in consonant articulation could play a role, given that Czech allows more complex consonant clusters. However, our analysis specifically focusing on consonant productions was based on the /pa-ta-ka/ task, which does not involve such clusters, making productions comparable. Conversely, variations in intonation patterns, such as the stronger stress in German, might impact results. To enhance the generalizability of findings and conclusively determine levodopa’s effect on speech, future studies should be conducted across multiple languages using consistent responder cut-offs.

## Methods

### Participants

Data of 119 individuals with idiopathic PD were included in the analysis which were collected during in-hospital stays between 12.02.2021 and 28.11.2023 in the Department of Neurology of the University Hospital Cologne. The retrospective data analysis was approved by the local ethics committee (protocol code: 23-1461-retro; date of approval: 6 December 2023). All participants provided written informed consent for their speech to be recorded and analyzed for research purposes. They were all speakers of German living in the Rhineland region close to the Benrather isogloss with at most a dialectal coloring but without a strong dialect. All have been diagnosed with PD according to the UK brain bank criteria^38^.

PwPDs’ speech and motor data were tested in two conditions, OFF and ON medication, by means of a levodopa challenge test. This test examines the effect of a standardized levodopa dosage on motor functions^39^. To achieve the OFF condition, PD medication was withdrawn for at least 12 h. For the ON condition, each participant received 200 mg soluble levodopa (2 × 100/25 mg levodopa/benserazide tablets). Data were collected in the OFF condition first, and second in the ON condition 30–40 min after levodopa intake.

Part III of the ‘Unified Parkinson’s disease ratings scale^40^ was used to monitor motor functions of all participants in both conditions. The total score was used to calculate the levodopa response of each individual^39^. According to the response, PwPD were divided into responders and non-responders as it has been done before by Tykalova et al.^10^. In contrast, we have chosen a cut-off of 30% (not 20%) based on the guidelines of the German Society for Neurology^41^. Non-responders were excluded from the final analysis in order to have a more uniform group and to reduce the heterogeneity associated with the disease in one parameter. We hoped to be able to explicitly investigate the levodopa effect, in which we were interested, for an initial analysis. Thus, the final number of PwPD is *n* = 78 (51 m, 27 f), aged between 33 and 81 years (*M* = 61.2 years, SD = 8.3).

### Elicitation of speech production data

Acoustic speech data were obtained by using a condenser microphone headset (AKG C 520, 44.1 kHz/16 bit) to keep the mouth-to-microphone distance constant. As suggested in the relevant literature^42,43^, a headset microphone was used and positioned in front of the speaker with a constant 5 cm distance from the lips at an angle of 45° away from the mouth. To avoid unwanted room reflections, we used an AKG C 520 microphone with a cardioid pattern (50-20.000 Hz) with a flat frequency response above 200 Hz for all acoustic recordings. The gain level was not adjusted between recording sessions and conditions. PwPD were placed in front of a TV screen that presented the German version of the speech task protocol of the “Dysarthria Analyzer” (dysan.cz)^23,44^ in a room with low ambient noise. Speech tasks included maximum phonation of the vowel /a/ in one breath at comfortable pitch and loudness for as long and steady as possible, fast syllable repetitions of /pa-ta-ka/ as quickly and accurate as possible in one breath to test oral diadochokinesis pattern (DDK), reading of a short text consisting of 80 words and a picture description task (monologue) to elicit free speech for approximately 90 seconds. Besides the picture description task, participants were asked to produce the tasks twice.

### Data processing and statistics

Speech features were automatically extracted by means of the MATLAB-written algorithm of the Dysarthria Analyzer^44^ providing a specific feature set. An overview of the extracted features per task is given in Supplementary Table S1. Values were averaged over repetitions per participant and medication condition.

Group comparisons of levodopa effects on speech features were tested by performing linear mixed-effects models using the “lme4 package”^45^ within the software R (version 4.2.2; R Core Team, 2023). 156 productions went into analysis. 78 were produced in the medication OFF condition and 78 in the medication ON condition. Linear mixed-effects models were built with treatment conditions, age and sex as predictor variables and random intercepts per participant. Main effects of treatment condition were validated by comparing the test model (with the critical predictor) to a reduced model (without the critical predictor) via likelihood-ratio tests. *P*-values are based on these comparisons. Post-hoc analyses were completed by using the “emmeans package”^46^ and applying the tukey method to correct for multiple comparisons if the main effect of the critical predictor was found significant. The level of significance was *p* < 0.05.

24 relevant acoustic speech features were selected based on mutual information for measuring speech motor functions as provided by the Dysarthria Analyzer^44^ (Supplementary Table S1). Speech features had to be significantly different between the medication conditions and significantly correlated with at least one of the two UPDRS scores to be considered for further analysis. The features for which one of both was the case were then combined into a single composite score. The composite score was built by summing up the values of each selected speech feature and then dividing the sum by the total number of included features. Thus, this composite score reflects an average of the features which is used as the potential speech biomarker for determining levodopa-related speech changes.

For feature validation, we followed the V3 framework of the Digital Medicine Society including analytical and clinical validation^24^. For analytical validation, we determined construct validity by correlating the composite score with an established measure of speech motor deterioration in PD, namely the speech item (item 18). Since each participant has one measurement in both medication conditions, a repeated-measure correlation analysis grouped by participant was performed using the “rmcorr” function in R to account for the non-independence of observations and also to avoid multiple testing^47^. For clinical validation, we also performed a repeated-measure correlation analysis between the composite score and our measure of motor deterioration, i.e. total UPDRS III score. The output of the correlations was proven by modeling the effects of medication status and participant variability using linear mixed-effects models to examine the effect of the UPDRS scores on the composite score. The model included subjects as random factor. Furthermore, the differences in composite scores between OFF and ON medication was evaluated by performing linear mixed models with treatment conditions, age and sex as predictor variables and random intercepts per participant.

For the machine learning analysis, age and sex were regressed out from the dataset using a linear mixed-effects model beforehand. Thus, for each speech feature, a linear mixed-effects model was built, using age and sex as predictor variables, and the residuals from these models constituted the regressed-out speech features. Support Vector Machine, Extra Trees, Random Forest, Linear and Decision Tree models were trained on this dataset to classify medication status. The performance of each model was evaluated using leave-one-group-out cross-validation, where one participant performing the tasks twice—once OFF medication and once ON medication—was considered a group. Additionally, feature selection was performed in each cross-validation loop using the “SelectKBest” function from the “sklearn” package in Python, with mutual information as the scoring method. This integration of feature selection within the cross-validation process ensures that the model is trained and validated on the most relevant features without data leakage. The best model was reported, based on the highest ROC AUC scores (Fig. 2). However, ROC AUC values of the other models can be found in the Supplementary Table S2. The model can be shared for validation upon reasonable request to the corresponding author.

## Supplementary information

Supplementary Material

## Data Availability

The datasets analyzed during the current study and the code supporting the study results are available upon request to the corresponding author.
