# Supplementary material for: Automatic speech analysis combined with machine learning reliably predicts the motor state in people with Parkinson’s disease
Source: NPJ Parkinsons Dis. 2025 May 2;11:105. doi: 10.1038/s41531-025-00959-4 (PMC12048546; doi:10.1038/s41531-025-00959-4)
Supplement: Supplementary file 1 — Supplementary Material [file 41531_2025_959_MOESM1_ESM.docx]

SUPPLEMENTARY MATERIAL

**Table S1:** Overview of extracted acoustic speech features per task. Table is adapted from Hlavnička*^28,29^*.

| *Task* | *Feature* | *Interpretation* |
| --- | --- | --- |
| *DDK* | *Voice onset time*  *(VOT)* | *Interval between the oral constriction release in a stop consonant and the start of following vocalic voicing. If increased there is a coordination deficit between laryngeal (phonatory) and supralaryngeal (articulatory) muscles resulting in a diminished capacity initiating voicing.* |
|  | *Diadochokinetic rate*  *(DDKR)* | *Median number of syllables spoken in a given time period. If decreased slower articulation rate.* |
|  | *Diadochokinetic irregularity*  *(DDKI)* | *Standard deviation of the measured durations between consecutive voice onsets. If increased the repetition rate is more irregular due to involuntary speech movements of speech, impaired timing or discoordination between phonatory and articulatory speech systems.* |
|  | *Vowel duration*  *(VD)* | *Mean duration of detected voiced intervals. If increased vowel durations are prolonged due to slower speech movements.* |
|  | *Standard deviation of power*  *(stdPWR)* | *Standard deviation of the loudness envelope computed on all voiced intervals. If increased there is an excessive variation in speech loudness due to involuntary movements of respiratory muscles.* |
| *MONOLOGUE* | *Rate of speech timing*  *(RST)* | *Rate of voiced, unvoiced and pause intervals. If decreased there is a reduced stream of voiced, unvoiced and pause intervals, typically caused by a reduced range of speech movements and/or decreased syllabic rate.* |
|  | *Duration of pause intervals*  *(DPI)* | *Median length of pause intervals. If increased there are difficulties in initiating speech and/or omission of short pauses.* |
|  | *Standard deviation of power*  *(stdPWR)* | *Standard deviation of the loudness envelope computed on all voiced intervals. If increased there is an excessive variation in speech loudness due to involuntary movements of respiratory muscles. If decreased there is a low variation of loudness associated with perceptive impression of monoloudness.* |
|  | *Standard deviation of fundamental frequency*  *(stdF0)* | *Standard deviation of detected modal F0 in semitones estimated via the median absolute deviation. If increased there is an excessive variation in pitch. If decreased there is a low variation of pitch associated with perceptive impression of monopitch.* |
| *PHONATION A* | *Standard deviation of power spectral density*  *(stdPSD)* | *The standard deviation was determined by averaging the standard deviations across 16 frequency bands that reflect power spectral density in a given window. If increased there are involuntary movements of articulators, preeminently tongue.* |
|  | *Maximum phonation time*  *(MPT)* | *Total duration of vowel phonation. If decreased this is an indicator for reduced control over respiratory and/or laryngeal muscles.* |
|  | *Standard deviation of fundamental frequency*  *(stdF0)* | *Standard deviation of detected modal F0 in semitones estimated via the median absolute deviation. If increased there is an excess variation of fundamental frequency due to involuntary movements of laryngeal muscles or loss of control over modulation by laryngeal muscles.* |
|  | *Jitter* | *The feature refers to the cycle-to-cycle variation of pitch periods. Median distance between adjacent maximal fundamental frequency peaks within a sliding window. was calculated. If increased there is temporal instability of glottal pulses which are associated with hoarse voice quality.* |
|  | *Shimmer* | *The feature refers to the cycle-to-cycle variation of intensity. Median distance between adjacent amplitude peaks within a sliding window. was calculated. If increased there is amplitude instability of glottal pulses which are associated with hoarse voice quality.* |
|  | *Harmonics-to-noise ratio*  *(HNR)* | *Ratio of harmonic to non-harmonic components in the speech signal. If decreased there is increased signal noise due to turbulent airflow in vocal folds indicating hoarseness.* |
|  | *Proportion of subharmonic intervals*  *(PSI)* | *Ratio between the total duration of subharmonic intervals per total duration of voicing. If increased vocal folds vibrate asymmetrically with alternating period, amplitude, or both which is associated with rough voice quality.* |
|  | *Location of subharmonic intervals*  *(LSI)* | *Initial time of the first detected subharmonic interval occurring in the course of phonation. If decreased vocal folds started subharmonic vibrations early in the course of the phonation. Vocal folds are either more prone to subharmonics or neuromuscular control of vocal folds is deteriorated.* |
|  | *Proportion of fundamental frequency tremor*  *(PF0T)* | *Frequency tremor was detected within each production in a sliding window with 2 seconds in length and 100 milliseconds step. If increased there is an abnormal depth of frequency tremor of vocal folds causing periodic changes of fundamental frequency.* |
|  | *Proportion of amplitude tremor*  *(PAT)* | *Amplitude tremor was detected within each production in a sliding window with 2 seconds in length and 100 milliseconds step. If increased there is an abnormal depth of amplitude tremor causing periodic changes of loudness.* |
| *TEXT* | *Rate of speech timing*  *(RST)* | *Rate of voiced, unvoiced and pause intervals. If decreased there is a reduced stream of voiced, unvoiced and pause intervals, typically caused by a reduced range of speech movements and/or decreased syllabic rate.* |
|  | *Duration of pause intervals*  *(DPI)* | *Median length of pause intervals. If increased there are difficulties in initiating speech and/or omission of short pauses.* |
|  | *Standard deviation of power*  *(stdPWR)* | *Standard deviation of the loudness envelope computed on all voiced intervals. If increased there is an excessive variation in speech loudness due to involuntary movements of respiratory muscles. If decreased there is a low variation of loudness associated with perceptive impression of monoloudness.* |
|  | *Standard deviation of fundamental frequency*  *(stdF0)* | *Standard deviation of detected modal F0 in semitones estimated via the median absolute deviation. If increased there is an excessive variation in pitch. If decreased there is a low variation of pitch associated with perceptive impression of monopitch.* |
|  | *Net speech rate*  *(NSR)* | *Total number of syllables divided by the total duration of speech. If increased, syllable rate is faster. If decreased, syllable rate is slower.* |

**Table S2:** ROC AUC values of all models trained on the data set.

| Model | Support vector machine | Random  forest | Linear  model | Decision  tree |
| --- | --- | --- | --- | --- |
| ROC AUC | 0.686 | 0.713 | 0.736 | 0.577 |
